# Super-resolution fluorescence microscopy by line-scanning with an unmodified two-photon microscope

**DOI:** 10.1098/rsta.2020.0300

**Published:** 2021-06-14

**Authors:** Christian Pilger, Jakub Pospíšil, Marcel Müller, Martin Ruoff, Martin Schütte, Heinrich Spiecker, Thomas Huser

**Affiliations:** ^1^ Biomolecular Photonics, Department of Physics, University of Bielefeld, Bielefeld, Germany; ^2^ LaVision BioTec GmbH, Astastraße 14, 33617 Bielefeld, Germany; ^3^ Department of Radioelectronics, Faculty of Electrical Engineering, Czech Technical University in Prague, Technická 2, 166 27 Prague 6, Czech Republic

**Keywords:** SIM, structured illumination microscopy, super-resolution optical microscopy, multi-photon fluorescence excitation, laser scanning fluorescence microscopy

## Abstract

Fluorescence-based microscopy as one of the standard tools in biomedical research benefits more and more from super-resolution methods, which offer enhanced spatial resolution allowing insights into new biological processes. A typical drawback of using these methods is the need for new, complex optical set-ups. This becomes even more significant when using two-photon fluorescence excitation, which offers deep tissue imaging and excellent z-sectioning. We show that the generation of striped-illumination patterns in two-photon laser scanning microscopy can readily be exploited for achieving optical super-resolution and contrast enhancement using open-source image reconstruction software. The special appeal of this approach is that even in the case of a commercial two-photon laser scanning microscope no optomechanical modifications are required to achieve this modality. Modifying the scanning software with a custom-written macro to address the scanning mirrors in combination with rapid intensity switching by an electro-optic modulator is sufficient to accomplish the acquisition of two-photon striped-illumination patterns on an sCMOS camera. We demonstrate and analyse the resulting resolution improvement by applying different recently published image resolution evaluation procedures to the reconstructed filtered widefield and super-resolved images.

This article is part of the Theo Murphy meeting issue ‘Super-resolution structured illumination microscopy (part 1)'.

## 1. Introduction

Super-resolution optical fluorescence microscopy is rapidly gaining significant interest in the biomedical sciences [1]. It permits the observation of biological objects and processes in their native environment on a length scale well below the diffraction limit. Although a large number of mechanisms have been developed to achieve optical super-resolution, the majority of them still require significant modifications to either the fluorescent probes or the instruments which are used to achieve super-resolution. In many cases, the need for fairly unique microscope set-ups will require users to invest in a new microscope if they want to participate in this new field of imaging. Even the implementation of super-resolution structured illumination microscopy (SR-SIM), one of the few methods that does not place special requirements on the optical probes, requires a rather complex optical system which uses two or more mutually coherent beams in order to create diffraction-limited interference patterns in the sample [2–4]. The known periodicity and orientation of these patterns can then be used to compute images with approximately twice the spatial resolution in all directions. This requires the acquisition of typically 9–15 raw image frames with different pattern orientations and phases per image plane. With the introduction of modern high-speed spatial light modulators, SR-SIM can now be conducted at speeds up to hundreds of super-resolved frames per second [5,6], and, with graphics processing unit (GPU) based image reconstruction, instant display of the super-resolved images is possible [7]. SR-SIM can, however, also be realized by highly focused laser beams in a variant called image scanning microscopy (ISM) [8] and similar approaches derived from this [9–11], and in parallel form by MSIM [12] or iSIM [13] and variations thereof [14,15]. Even multi-photon fluorescence excitation and nonlinear optical microscopies have been realized in this way [16,17]. Although these instruments are still complex and rather specialized, this clearly demonstrates that it should, in principle, be possible to simplify this form of SR-SIM even further and to achieve super-resolution microscopy even with unmodified laser scanning microscopes.

Here, we investigate the realization of SR-SIM with a standard, commercial two-photon laser scanning microscope (2P-LSM), which requires just the addition of an sCMOS camera (which has previously been used in high-resolution 2P-LSM applications [18]) and a customized scanning protocol, which we refer to as ‘striped illumination'. We find that with these minor additions, it is possible to apply already existing and open-source SR-SIM image reconstruction software tools to 2P-LSM raw images and readily enhance both resolution and contrast in two-photon fluorescence microscopy. This provides a rather straightforward implementation of enhanced capabilities for existing 2P-LSM set-ups, which is quite independent of the microscopy platform used. In the following, we refer to data which were acquired with a specific commercial 2p-LSM set-up. The customized scanning protocol can, however, also easily be replicated e.g. in the popular 2P-LSM scanning control software ScanImage, an earlier version of which is still available through open access [19]. There, the required separation between vertical scan lines as well as the scan angle can be chosen directly within the graphical user interface of the software. In this paper, we demonstrate the process of raw image data collection by 2P-LSM striped illumination and the reconstruction of super-resolved images by two popular open-source image reconstruction packages, fairSIM and SIM Toolbox [20,21]. We also analyse the degree to which the spatial resolution is improved by applying three different image resolution estimation tools, full width at half maximum (FWHM) estimation, image decorrelation analysis [22] and circular average power spectral density analysis [23].

## 2. Materials and methods

**Figure 1.**
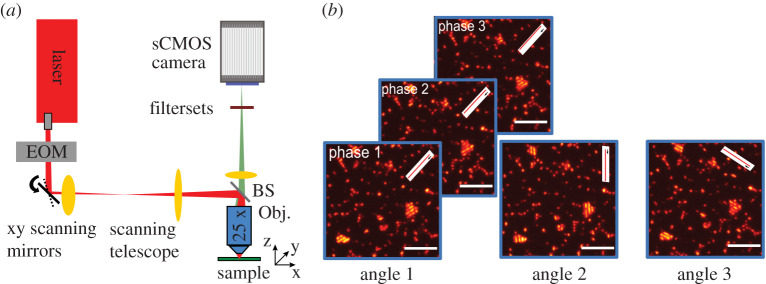
(*a*) A 140fs pulsed laser at approximately 800 nm is focused into the sample by an objective lens (Obj) and raster scanned by fast galvanometric scanning mirrors. A short pass beam splitter (BS) is separating the excitation laser light from the two-photon excited fluorescence. The fluorescence signal is focused by a tube lens onto an sCMOS camera mounted onto the microscope's body. An electro-optic modulator (EOM) is placed in the laser beam path and performs rapid intensity modulation. Specifically adapted scan software generates a striped-illumination pattern addressing the EOM, galvo-mirrors and the camera trigger. (*b*) Exemplary raw dataset showcasing the image acquisition procedure on a sample of fluorescent micro beads with 1 µm diameter. The phase of the illumination pattern is shifted three times by a step size of π/3, in the next step, the pattern is rotated by ±π/3 and again the pattern is shifted for generating in total nine raw images for the reconstruction process. Note that the displayed pattern is very coarse to illustrate the scanning process. Scale bar indicates 5 µm. (Online version in colour.)

In this report, a standard two-photon laser scanning microscope (TrimScope II, LaVison BioTec) equipped with a wavelength-tunable femtosecond laser source (Chameleon Ultra I, Coherent) with approximately 140 fs pulse length is used for generating striped-illumination patterns on the sample (figure 1 for a schematic of the optical set-up). In 2P-LSM, the sample is usually raster scanned by rapidly shifting the laser focus in a line-by-line manner across the field of view. For this purpose, fast galvanometric xy-scanning mirrors are addressed by the output of digital-to-analogue converters. The emitted fluorescence signal, collected by the same objective lens, is then directed onto a photomultiplier tube (PMT), whose electronic signal is sampled by an analogue-to-digital converter. This fluorescence intensity value is temporally matched to the focal position in the sample by the scanning program, which generates the final two-photon excited fluorescence image. Common to a large number of 2P-LSM systems is that they are also often equipped with a fast electro-optical modulator (EOM), which can be used as a pulse-picker for reducing the laser's repetition rate, or to blank out or lower the laser intensity as needed in various applications depending on the sample of interest and the needs of the operator.

Due to the process of two-photon excitation of fluorescent dyes, the spatial resolution of 2P-LSM is readily enhanced compared to standard fluorescent microscopes: since two pump photons are required to be simultaneously absorbed by the sample in the excitation process, the point spread function (PSF) is effectively squared, and thus slimmer than in one-photon excitation. This effect is rather significant for the axial resolution of the microscope leading to a unique z-sectioning capability without the need for pinholes in the detection path, which otherwise reduce the signal intensity in one-photon laser scanning microscopes. The near-infrared excitation wavelengths also permit deep tissue imaging. To further improve the spatial resolution of this already powerful tool, we devised a way of taking advantage of the principle of structured illumination microscopy for two-photon excitation microscopy in a way that could easily be implemented on most 2P-LSM systems. Here, a striped-illumination pattern with a line spacing approaching the highest supported spatial frequency of the microscope's optical transfer function (OTF) is generated in the focal plane of the sample by a customized scan process. In SR-SIM, this pattern is usually generated by interfering two mutually coherent laser beams, and the resulting pattern is then laterally shifted in its phase across the sample in at least three or more steps. In a next step, the illumination pattern is rotated by π/3 and −π/3, where for each rotation angle the phase of the pattern is then again shifted as described before (figure 1*b* for an example of this process). If the line spacing is set close to the maximum transmitted spatial frequency, higher harmonics of the pattern are blocked. Hence, an image series of (at least) nine images is acquired with three-phase steps for each rotation angle of the pattern in order to obtain sufficient information about the sample for the successful reconstruction of a super-resolved image. In this way, a known spatial frequency is introduced into the images, which mixes with higher, undetectable frequencies of the sample lying beyond the OTF support of the set-up [2]. This can be exploited in a post-processing step, where the signal is first unmixed in a band separation step, followed by shifting the bands to their original positions in frequency space, which is given by the orientation and spatial frequency of the illumination pattern. For a sinusoidal illumination pattern, three-phase steps are needed to fully determine the band separation. If higher harmonics are contained in the illumination pattern, two additional phase steps are needed per additional harmonic. For the successful reconstruction, the exact angle and phase of the introduced illumination pattern have to be precisely determined, which can be achieved by cross-correlation of the frequency content beyond the native OTF support of the particular set-up [2,20,24]. Several structured illumination microscopy reconstruction tools have been developed in the last decade, including the open-source ImageJ plugin fairSIM [20] and the MATLAB toolbox SIMToolbox [21]. fairSIM performs standard Gustaffsson SIM reconstruction based on decomposing and shifting of the spectral components [2]. SIMToolbox, on the other hand, exploits the maximum *a posteriori* probability SIM (MAP-SIM) method [25], which does not decompose spectral components, but works with data in real space. This method is based on combining the maximum *a posteriori* probability (MAP) estimation (for lateral resolution improvement) and homodyne detection (for optical sectioning). To obtain MAP-SIM images, SIMToolbox merges the low-resolution homodyne detection and high-resolution MAP images in the frequency domain. Furthermore, MAP-SIM does not require the precise knowledge of the illumination pattern position in each image within an image sequence. Both reconstruction methods are able to process data from all SR-SIM microscope set-ups using standard interference-based illumination patterns as introduced by Gustaffson, for instance, raw data from all currently available commercial platforms (Nikon, Zeiss, GE Healthcare), as well as many custom-built set-ups [26–28].

Transferring the concept of structured illumination to two-photon laser scanning microscopy requires the replacement of the PMT, which is a point-detector, by an sCMOS camera acting as a spatial image detector. For this purpose, the camera trigger and readout are synchronized to the movement of the scan mirrors and the time they need to sweep the laser beam across the full field of view. The camera used in our experiments (Neo 5.5, Andor) has a physical pixel size of 6.5 µm which, in combination with a 300 mm tube lens and the 60x 1,42NA oil objective lens (Olympus, ApoN, effective focal length 3 mm) leads to an image pixel size of 66 nm, which is set in this way to provide slightly oversampled detection, in line with typical SIM system designs.

The illumination pattern is generated by using the EOM as fast intensity switch, which shutters the excitation laser when the scan mirrors address a sample area which is not part of the desired pattern.

The pattern spacing and also the angle of rotation can easily be changed and optimized for a specific objective lens by an instrument-specific custom-written Python-based scanning protocol. The microscope's scan control software (ImSpector, LaVision BioTec GmbH) allows for this custom-specific extension of scan protocols by using Python macros based on an instrument-specific control module. With this macro, the line separation between adjacent scan lines can be set, as well as the angle that defines the fast axis of the scan system. The same functionality is e.g. readily built into the popular scan control software ScanImage [19], early open access versions of which are still available and which we e.g. use to operate custom-build coherent Raman scattering microscopes [29].

For the acquisition of a single-raw image, the EOM first shutters the laser beam and the scanning mirrors are moved to the start of the first illuminated line of the overall pattern. Then, the EOM opens the shutter and the camera trigger is set to start the image acquisition. The scan mirrors move the laser focus along the illuminated line, then the EOM shutters the laser beam, again. The scan mirrors move to the start of the next illuminated line and the EOM opens the shutter, again. This protocol is repeated until the desired illumination pattern is completed and the camera image acquisition process is stopped. To collect the next raw image the entire path of the line pattern is translated along the slow scan axis by a constant offset corresponding to the phase step of the pattern. In order to change the rotation angle of the pattern, the angle parameter available in the scan control software is changed, which causes the scan mirrors to rotate the fast axis of the image scan (figure 1*b*). The precision of the galvanometric scan mirror movement was not a limitation in these experiments, as they were well calibrated at the beginning of the experiment in order to avoid any scanning artefacts due to creep or oscillations along the pattern axis. The exact pattern spacing was determined and optimized in a separate experiment using a dye solution or a monolayer of fluorescent microbeads (beads with diameters of 200 nm and 1 µm were used). Here, two main parameters are optimized: On one hand the pattern spacing is optimized as much as possible such that the line pattern can still be detected in the camera image in order to achieve the highest possible resolution enhancement (i.e. the pattern frequency still needs to be transmitted by the OTF of the optical system). On the other hand, the modulation depth of the pattern is decreasing in the higher frequency range due to the decreasing OTF support of the system as well as due to scattering effects, which also depend on the sample of interest. The modulation depth (controlled by the line spacing) is, thus, kept above a value of 0.5 in order to permit successful image reconstruction. In these particular experiments, we found the optimal pattern shift to be 70 nm per phase setting for the 60 x objective lens (1,42NA)), as well as 450 nm using the 25x objective lens (1,1NA), respectively.

**Figure 2.**
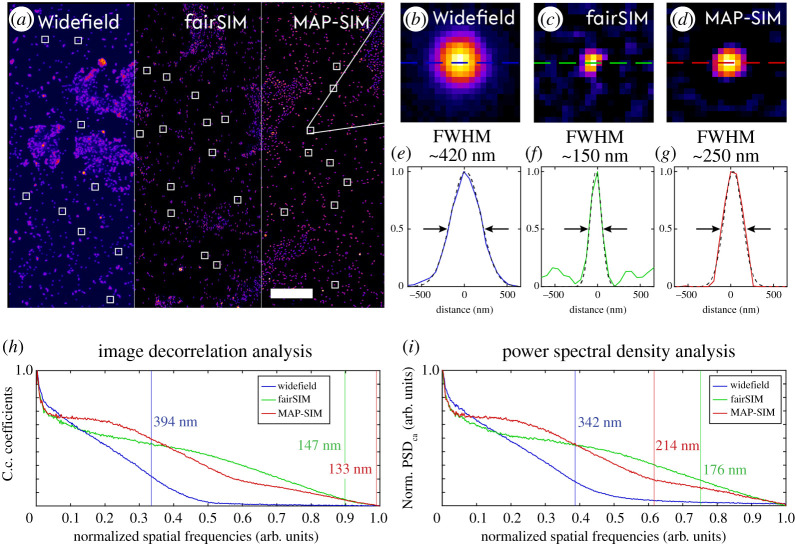
Measurements to estimate the spatial resolution conducted on a fluorescent bead sample. (*a*) Images of a fluorescent bead sample separated by the corresponding reconstruction approach: low-resolution widefield, fairSIM and MAP-SIM. White squares indicate randomly chosen ROIs containing individual fluorescent beads used for full width at half maximum (FWHM) calculation (all 31 ROIs were used for each imaging technique). (*b*), (*c*) and (*d*) Show one ROI with an individual fluorescent bead reconstructed by widefield, fairSIM and MAP-SIM, respectively. Dashed lines correspond to line profiles in (*e*), (*f*) and (*d*). Results of decorrelation analysis and circular average power spectral density analysis are shown in (*h*) and (*i*). Vertical lines indicate cut-off frequencies for each imaging technique. The spatial frequency axes are normalized by the sampling rate $f_{s}=1/2\times \text{pps}=7.57\;\mu {\text{m}}^{-1}$ (pps = projected pixel size). Scale bars are 10 µm. (Online version in colour.)

**Figure 3.**
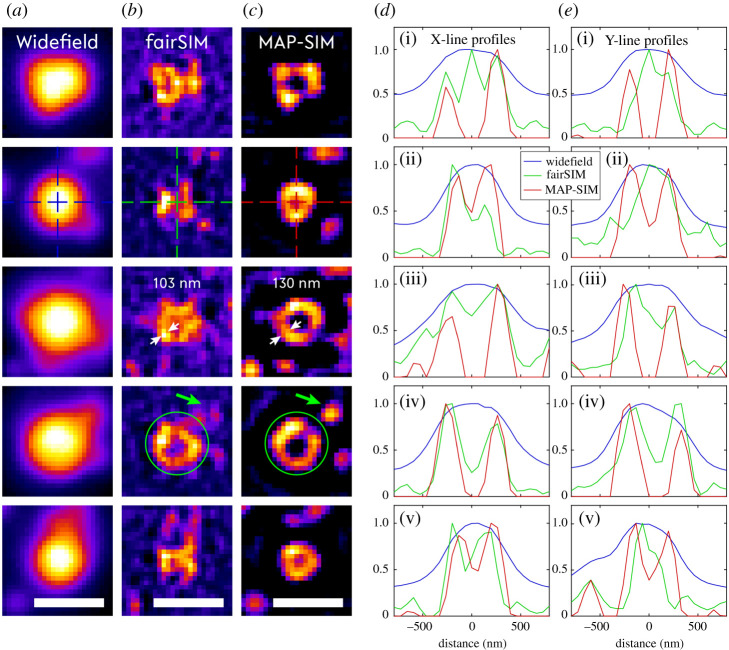
Comparison of two SIM image reconstruction techniques. Each row shows manually chosen ROIs from figure 2. The three columns correspond to the different image reconstruction techniques. From left to right: widefield, fairSIM and MAP-SIM, respectively. Dashed crosses indicate the X- and Y-line profiles. The white arrows in the third row indicate the resolution improvement in the lateral direction. The green arrow and the circle in the fourth row point out the individual bead and the group of the beads, respectively. Scale bars are 1 µm. (Online version in colour.)

In an initial experiment, the optimal pattern spacing was determined by reducing the line spacing in a series of images of a fluorescent bead sample (bead diameter *Ø* = 200 nm, TetraSpeck microspheres, ThermoFisher). The fluorescent beads were prepared by diluting the stock solution by a factor of 1:5 and allowing a 60 µl drop to dry on a #1.5 glass coverslip by turning it upside down (hanging droplet technique) to generate a monolayer of fluorescent beads. Reconstruction of the super-resolved image acquired by the striped-illumination approach was performed by fairSIM and SIMToolbox. The fairSIM reconstruction used Richardson-Lucy iterative deconvolution-based filtering [30] with 15 iterations. The apodization cut-off was set to 1.2. Two-photon fluorescence excitation readily leads to high contrast z-sectioning during image acquisition. Therefore, in SIMToolbox the MAP-SIM spectral merging parameter was set to 0.98, which corresponds to a merger of 98% of the MAP estimation and 2% of the homodyne detection. The raw dataset containing 9 images was averaged and used as the widefield image for comparison to the reconstructed images (see figures 2 and 3).

## 3. Results and discussion

We first imaged a fluorescent bead sample (bead diameter 200 nm, prepared as described in Materials and methods) in order to determine the degree to which the spatial resolution could be enhanced by our striped illumination approach. Widefield fluorescence and reconstructed super-resolved images (using fairSIM and SIMToolbox) are shown in figure 2. We used three different resolution estimation methods, including full width at half maximum (FWHM) estimation, image decorrelation analysis (imDecorr) [22] and circular average power spectral density (PSDca) analysis [23] in order to obtain a mostly unbiased determination of the gain in spatial resolution provided by our illumination scheme. The filtered widefield image is also shown in figure 2 in order to assess the enhancement of contrast by the necessary filtering steps during the reconstruction process. Richardson-Lucy deconvolution filtering was applied to the widefield image. In the reconstructed images (figure 2*c,d*), the contrast and resolution are clearly enhanced, which can also be seen by examining the corresponding line plots (figure 2*e*, *f*, and *g*). These plots illustrate the FWHM measurement of an individual fluorescent bead. A Gaussian fit to the single bead data is used to determine FHWM values. The final resolution estimate above each plot is determined as the average of FWHMs calculated for 31 individual beads (the white squares in figure 2*a*). Results of imDecorr and PSDca analysis are shown in figure 2*h*,*i*. The spatial frequency axes are normalized by the sampling rate (given by the projected pixel size in µm) equal to $f_{s}=1/2\times \text{pps}=7.57\;\mu {\text{m}}^{-1}$ (pps = projected pixel size). Furthermore, five regions of interest are magnified in figure 3 showing a group of fluorescent beads (i.e. up to six single beads), which appear to be arranged in the shape of a circle. This group of beads cannot be resolved in the widefield images whereas the reconstructed images reveal the gaps between individual beads making up the bead layer, which are also visible in the line plots by a clear drop in intensity.

Note that patterns generated by scanning the laser focus across the sample while the camera is in exposure mode result in a significantly different pattern compared to those generated by laser beam interference. Striped illumination results in a pattern which does not exhibit a truly sinusoidal intensity distribution of the individual lines of the pattern. Instead, the rectangular-shaped scan function of the scanning mirrors is smoothened by the OTF support of the objective lens leading to an illumination pattern which also contains higher harmonic frequencies of the fundamental pattern. This feature can be useful by further extending the accessible frequency range and is analysed by the SIMToolbox, which is designed for exploiting higher harmonics in the illumination pattern. This does, however, require the pattern to be sufficiently coarse so that the higher harmonics are not cut-off by the resolution limit of the instrument. In figure 3, a comparison is performed on the dataset of the bead sample contrasting fairSIM-based reconstruction to MAP-SIM, while both methods are operated within their available parameter settings set for their best performance.

The image decorrelation analysis introduced by Descloux *et al*. calculates the decorrelation of the image under investigation with its high-pass filtered version in several iteration steps, where the high-pass cut-off frequency is changed [22]. The algorithm looks for the cut-off frequency of the high-pass filter where the filtered image shows any correlation with the original image. The higher frequencies above the cut-off contain purely noise. Note that in the case of the MAP-SIM image reconstruction, which effectively suppresses the noise in the image, the decorrelation analysis reaches an apparently better result due to the low background noise level, but the amount of high-frequency information of the sample is still very similar to that produced by fairSIM reconstruction (figure 3). Based on these analyses, both FWHM and imDecorr would indicate an overall resolution improvement of better than 2 x, which is not realistic for a process that is, in essence, similar to ISM or rescan confocal. The PSDca analysis results in a roughly 1.6 x improved resolution for the MAP-SIM reconstructed image and approximately 1.9 x improved spatial resolution for the fairSIM reconstructed image, i.e. much better in line with super-resolution microscopy approaches using highly focused laser beams.

**Figure 4.**
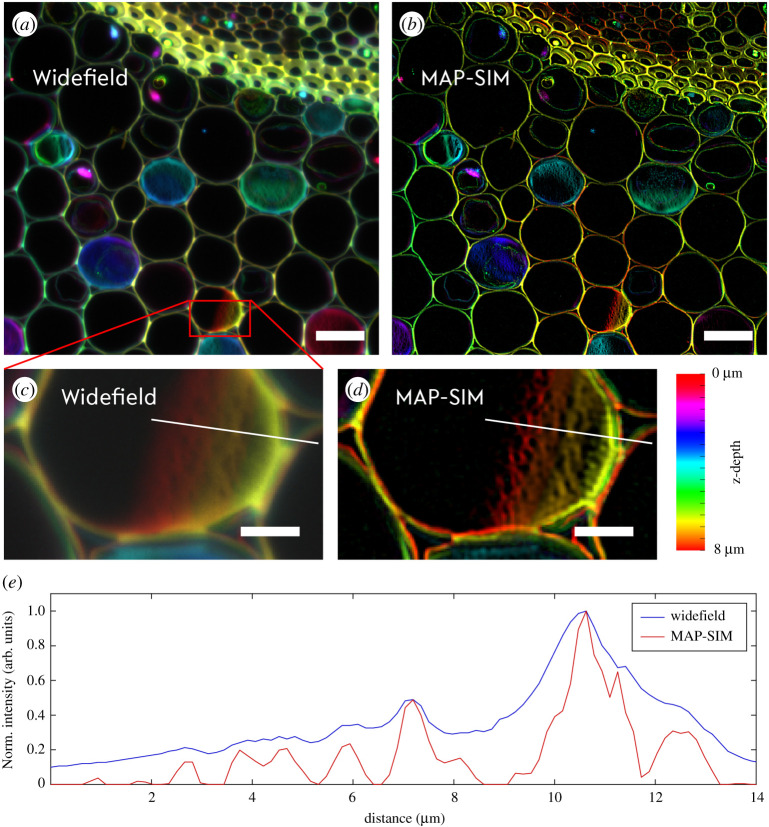
Auto-fluorescence of a Convalaria majalis sample excited by 800 nm laser light while striped-illumination patterns are applied in a series of nine images using a 25 x water-immersion objective lens. Sequentially the z-focus is shifted with a step size of 0,5 µm across a full axial range of 8 µm, the z-information is false-colour coded. By comparing the widefield image to the reconstructed image using MAP-SIM, the enhanced spatial resolution and contrast are quite apparent. Subfigure (*e*) shows the normalized line profiles from (*c*) and (*d*). The scale bar is 20 µm in the upper row and 5 µm in the inset. (Online version in colour.)

In biomedical applications of two-photon laser scanning microscopy, a larger field of view as well as a long working distance is often required, which is typically realized by using a 25 x water-immersion objective lens with a high numerical aperture (NA). In order to investigate the capabilities of the striped-illumination approach under these conditions, we imaged a thin section of a Convalaria majalis sample using a 25 x, NA1,1 objective lens (Nikon, working distance 2 mm). Here, auto-fluorescence in the sample is excited by two-photon excitation and a z-scan through the sample with a sample thickness of 8 µm was collected. The z-scan was performed using a micro-motor drive built into the TrimScope II. The spacing of the illumination pattern was again adapted to the changed OTF of the microscope, i.e. it was selected to be coarser due to the lower NA of the objective lens. For each axial plane, nine images with shifted illumination patterns were acquired, then the focus was moved by 0.5 µm across the total vertical range of 8 µm. The z-image stack was reconstructed by MAP-SIM (figure 4), then false-colour coding stretching from red to orange was applied to the image in order to encode for the best vertical focal positions. The zoomed inset to figure 4 shows the enhanced contrast and resolution also in the axial dimension, where small features become readily discriminable.

**Figure 5.**
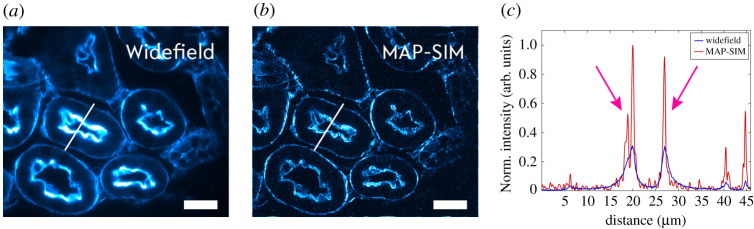
The two-photon excited fluorescence of Alexa Fluor 568 of a murine kidney tissue section (#3, ThermoFisher) is imaged with a 60 x oil immersion objective lens. The striped-illumination raw images are reconstructed by MAP-SIM leading to enhanced resolution and contrast also visible in the line plot (red line), especially at the cellular borders. The scale bar is 25 µm. (Online version in colour.)

To demonstrate yet another biomedical application, a murine kidney tissue section (FluoCells prepared Slide #3, ThermoFisher) stained with three fluorescent dyes (Alexa Fluor 488 WGA, Alexa Fluor 568 and DAPI) was investigated by setting the femtosecond laser wavelength to 800 nm in order to excite the Alexa Fluor 568 dye. Here, the sample was scanned by using the 60 x, NA1.42 objective lens, again, together with its previously determined corresponding line spacing. Alexa Fluor 568 stains the filamentous actin prevalent in the glomeruli and the brush border in this sample. In front of the sCMOS camera, a 595/40BP (Chroma, D595/40x) bandpass filter was installed to isolate the desired signal which peaks at approximately 603 nm. Image reconstruction was performed in MAP-SIM (figure 5). The averaged widefield image and the reconstructed images are compared in figure 5, and the line plot (indicated by a red line) again confirms the improved resolution of the reconstructed image compared to the widefield image. As can be seen in figure 5, in addition to the enhanced image contrast, significantly steeper edges are also visible at the cellular borders (pink arrows in the line plot).

## 4. Conclusion

We demonstrated the implementation of a striped-illumination approach in two-photon laser scanning fluorescence microscopy by using a custom-written, Python-based macro which controls the galvanometric scan mirrors in combination with rapid intensity switching by an EOM. The special appeal of this approach is the minimal effort in additional hardware needed to exploit the structured illumination technique. The optomechanics of the microscope remains essentially unmodified with the simple addition of an sCMOS camera and a suitable tube lens, which was already used in previous high-resolution imaging applications [18]. We compared different image reconstruction methods for the final post-processing step, showcasing the potential of this method by applying it to several different samples ranging from fluorescent beads to commercially available stained murine kidney tissue samples, and a freshly cut auto-fluorescent plant section. This highlights the versatility and robustness of our approach even when using different optical magnifications, larger fields of view and lower NA by changing the microscope objective lens, as well as highly scattering samples, which are often difficult, if not impossible, to image with interference pattern-based SR-SIM. Compared to other implementations of one- and two-photon SIM, such as line-scan, rescan confocal or image scanning microscopy [11,31–33], no complicated and expensive additional optical components or additional maintenance are required—resulting in a very user-friendly operation, which could even be implemented and used by non-experts in the field.

## Data accessibility

The original raw data obtained by stripe scanning of the various samples used to demonstrate super-resolution SIM-like imaging with the unmodified TriMScope II are openly accessible through zenodo.org: https://doi.org/10.5281/zenodo.4080909. The most recent versions of the Open Source, Open Access image reconstruction methods used to process the data can be found at: https://github.com/fairSIM and https://simtoolbox.github.io/.
